# ResA^3^: A Web Tool for Resampling Analysis of Arbitrary Annotations

**DOI:** 10.1371/journal.pone.0053743

**Published:** 2013-01-28

**Authors:** Aaron Ruhs, Franz Cemic, Thomas Braun, Marcus Krüger

**Affiliations:** 1 Biomolecular Mass Spectrometry and Cardiac Development and Remodeling, Max Planck Institute for Heart and Lung Research, Bad Nauheim, Germany; 2 Institute for Biochemical Engineering and Analytics, University of Applied Sciences, Giessen, Germany; Saint Louis University, United States of America

## Abstract

Resampling algorithms provide an empirical, non-parametric approach to determine the statistical significance of annotations in different experimental settings. ResA^3^ (Resampling Analysis of Arbitrary Annotations, short: ResA) is a novel tool to facilitate the analysis of enrichment and regulation of annotations deposited in various online resources such as KEGG, Gene Ontology and Pfam or any kind of classification. Results are presented in readily accessible navigable table views together with relevant information for statistical inference. The tool is able to analyze multiple types of annotations in a single run and includes a Gene Ontology annotation feature. We successfully tested ResA using a dataset obtained by measuring incorporation rates of stable isotopes into proteins in intact animals. ResA complements existing tools and will help to evaluate the increasing number of large-scale transcriptomics and proteomics datasets (resa.mpi-bn.mpg.de).

## Introduction

Gene and protein annotations like Gene Ontology, KEGG or Pfam provide a systematic approach to classify protein function and localization. The statistical analysis of these gene annotations allows deep insight into regulatory circuits between functionally and spatially related groups of genes. To identify over-represented groups of genes and proteins from a large-scale dataset, a target set based on fold-change or some statistical value is constructed to distinguish between regulated and non-regulated candidates in most analyses. For example, tools like GOrilla, GoMiner and Catmap [1], [2], [3] use separate target and background sets to calculate enriched GO terms, or use a ranked gene list without experimental values. However, arbitrary cutoffs generate a bias and information in the dataset could be lost. Also, ranked lists lack any information about the type of distributions. Thus, for a more impartial analysis, random permutation approaches independent of cutoffs, were developed. ErmineJ [4], a tool providing a microarray focused permutation-based analysis, will be discussed later.

**Figure 1:**
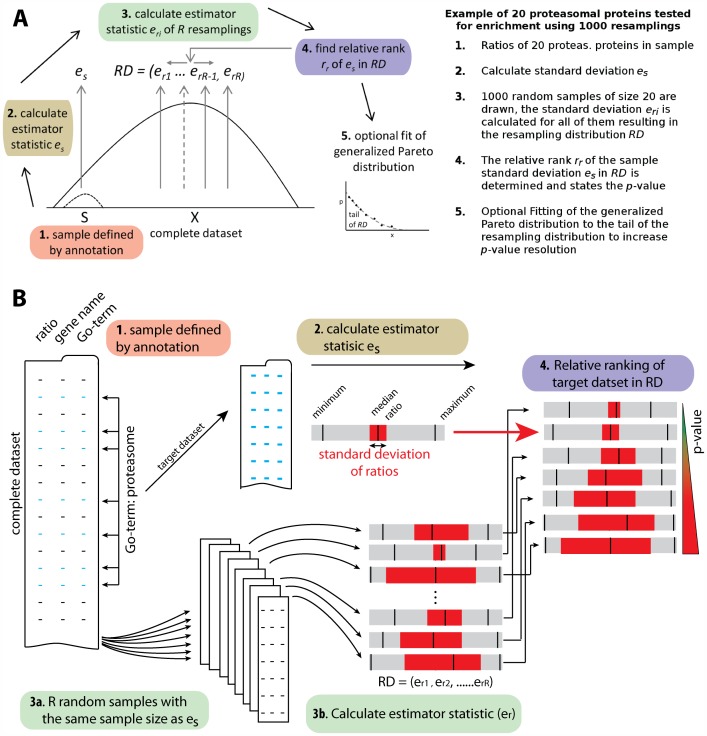
Overview of the resampling procedure. (**A**) The resampling procedure is done in five steps. 1. The *m* experimental values, which are associated with a given term of annotation, are collected. 2. The estimator statistic (*e_s_*) of this sample is calculated. 3. The resampling procedure samples the complete data set *R* times (*m* out of *n*) by picking *m* elements randomly with replacement. The estimator statistic is evaluated for each sample (*r_s_*) and stored in the empirical resampling distribution (*RD*). 4. The relative rank (*r_r_*) of *e_s_* in *RD* is determined. 5. Optional fitting of the generalized Pareto distribution to the tail of *RD*. (**B**) Graphical illustration of the resampling procedure and relative ranking of the standard deviation.

Here, we present ResA, a universal web tool designed to determine the statistical significance of sample distributions defined by annotation in genomic and proteomic data sets. Samples of experimental values linked to an annotation were evaluated for the significance of a statistical property (estimator) such as standard deviation (SD), coefficient of variation (CV) or deviation of the mean. ResA allows analysis of the enrichment and regulation of terms associated with protein complexes, function and other classifications. Significance is estimated by the application of a resampling algorithm. The algorithm estimates empirically the significance of a statistic of a selected set of experimental values. This is done by repetitive and random collection of samples of the same size from the complete dataset. For example, gene ontology analysis revealed that 20 proteins from the whole dataset belong to a proteasomal term and the estimator statistic (i.e. SD) of the given experimental values (i.e. incorporation rate of stable isotopes) is calculated. ResA compares this statistic to that of 1000 randomly selected sets of the same size from the whole dataset. If the random sample statistics are mainly less extreme compared to the proteasomal set, the determined *p*-value will be low. (**Figure 1A, B**). In addition, ResA is not limited to common annotations and is able to handle any custom type annotation. Moreover, it provides a feature for full and slim Gene Ontology annotation of nine different organisms based on gene names and UniProt identifiers using the UniProt-GOA [5]. ResA has no limitations with respect to type of distribution, since the resampling approach accounts inherently for the distribution of the underlying population. Here we show that ResA is able to test for significantly regulated terms and is capable of enrichment analysis within the complete data set as demonstrated by the analysis of ^13^C_6_-Lysine (Lys6) incorporation rate experiments in living animals. In addition to the *p*-value, the false discovery rate (FDR) is empirically determined taking into account dependencies within the data set [**6**]. Taken together the tool provides unbiased extreme value (regulation) and enrichment analysis without choosing a cutoff or interval to define the target data set.

### Design and Implementation

The ResA algorithm was implemented using Python 2.5.2 with SciPy 0.6.0 and R 2.6.2. The web interface was implemented using Python, HTML, CSS and Java Script on an Apache web server running the mod_python module. **Figure 1A, B** illustrates the general flow of the resampling procedure. After parameter setup, data upload, and optional Gene Ontology annotation the resampling analysis on the chosen estimator is applied to each sample defined by the annotations provided.

The *m* experimental values, which are associated with a given term of annotation (i.e. GO-term), are collected and the estimator statistic (*e_s_*) of this sample is calculated. The resampling procedure samples the complete data set *R* times (*m* out of *n*) by picking *m* elements randomly with replacement. The estimator statistic is evaluated for each sample (*r_s_*) and stored in the empirical resampling distribution (*RD*). After *R* iterations the *RD* is sorted and the relative rank (*r_r_*) of *e_s_* is determined. Of note, the relative rank is to a close approximation equal to the probability of obtaining the same or a more extreme value for *e_s_* by chance. Therefore, the relative rank gives the type I error probability, which reflects the significance level of the target set. To increase the resolution, linear interpolation between ranks and optional fitting of the generalized Pareto distribution to the upper and lower 2% of the *RD* is done by default using the R-package fExtremes (**Figure S1 and S2**). To increase the speed of analysis, the empirical resampling distributions are reused with samples of equal size *m* for the estimation of type 1 error probability. We estimate the false discovery rate (FDR) using permutations of the dataset serving as *H_0_* distribution while retaining the interdependence of the underlying data. Specifically, this is done for each *p*-value by dividing its rank in the *H_0_* distribution (*rpH_0_*) by its corresponding rank in the *H_1_* distribution (*rpH_1_*) [6]. A minimum of 1000 *p*-values, being a multiple of *n,* based on the H_0_ distribution are generated to provide sufficient resolution of the FDR estimation.

### Program Usage and Settings

**Figure 2:**
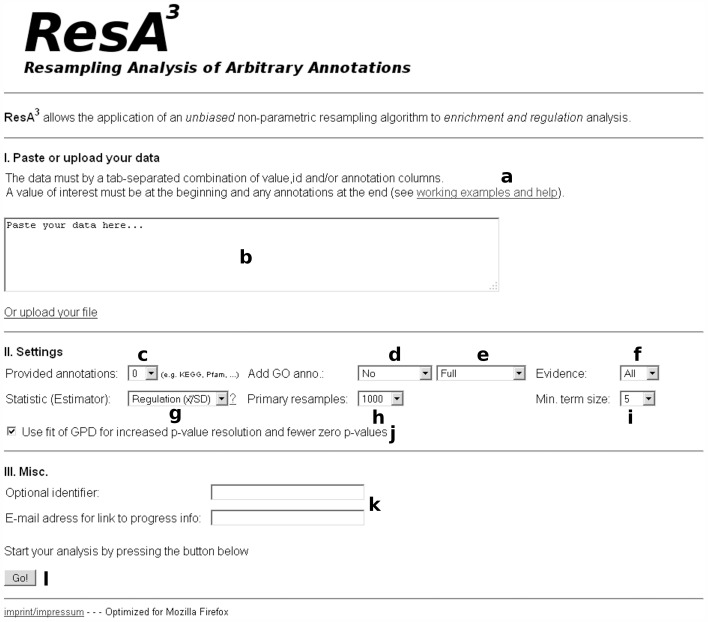
Screenshot of the user interface. The overview shows the first layer of the ResA user interface. All input fields (**a**–**l**) are explained in the text.

**Figure 3:**
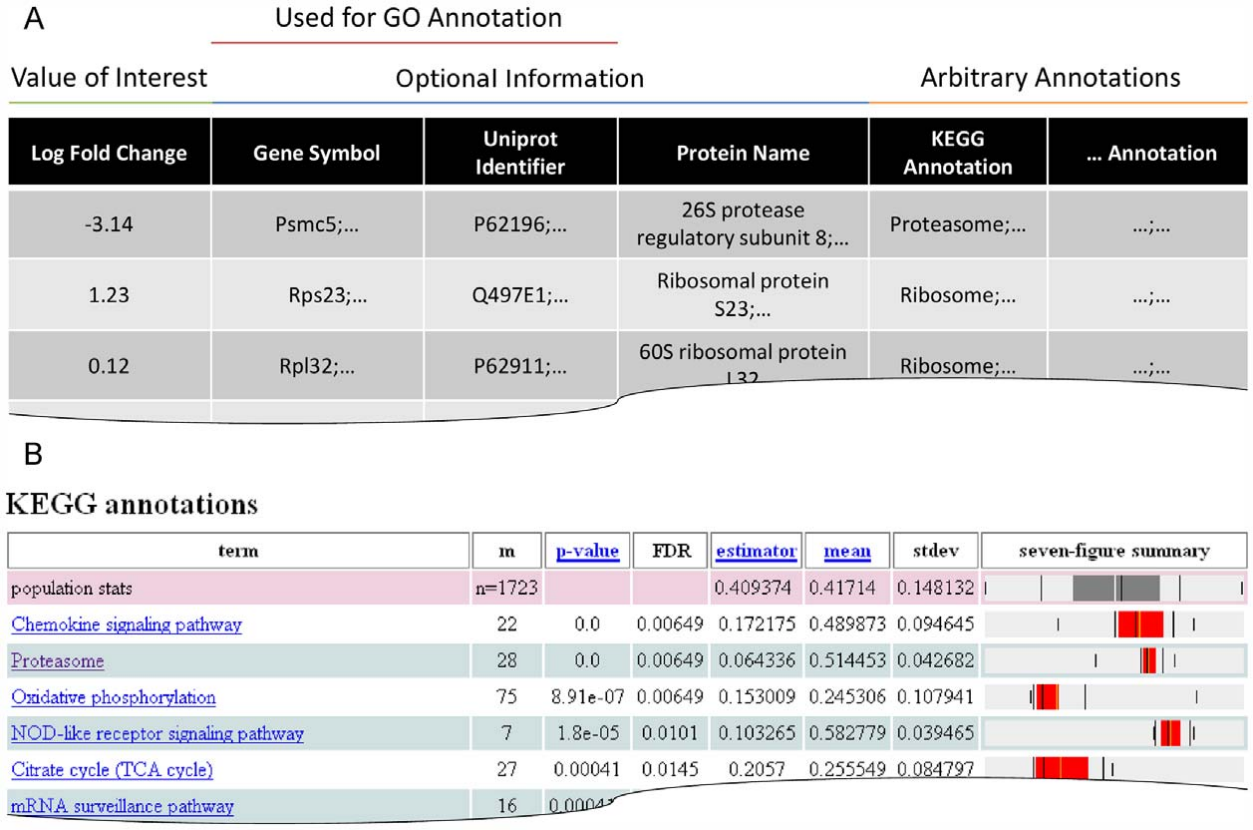
Representative results of an enrichment analysis. (**A**) Shows the format of the input data. The general order of the input data is: Value of interest, optional columns for gene names or UniProt for GO annotation) and annotations. If annotations are provided, the count of columns must be set in the spin box (**Figure 2c**). Multiple identifiers and annotations within columns must be separated by semicolons. (**B**) Representative output of KEGG-terms after enrichment analysis. It contains, along with the *p*- and *FDR*-values basic sample statistics and a seven-figure summary. The summary diagram contains the minimum and maximum (outer whiskers), p_10_-percentile and p_90_-percentile (inner whiskers), first and third quartiles (box), median (black line in box) and mean (light line in box).

Datasets can be inserted or uploaded to the web interface (**Figure 2b**). Data must be tab separated and formatted as shown in **Figure 3A**. They could include gene symbols and UniProt identifiers, which are used for the Gene Ontology annotation feature. To annotate a dataset which contains gene symbols and/or UniProt identifiers, the appropriate organism can be set (**Figure 2d**) and the user can choose between full or slim GO annotation (**Figure 2e**) and full or experimental evidence (**Figure 2f**). The annotation is based on the gene and UniProt identifiers provided by UniProt-GOA and will be updated monthly.

It is essential that the first column contains the experimental value (log fold-change, isotope incorporation, intensity, etc.). Titles of the annotation columns are used to discriminate between different types of annotation in the results. If a pasted dataset already contains annotations such as KEGG and Pfam, these terms must reside in tab separated and titled columns (**Figure 3A** and online help). Annotations need to be located in the last columns. Importantly, semi-colons must separate multiple identifiers or terms in one column. For example, multiple identifiers are common for protein groups of mass spectrometric data and annotation will be done for all entries. Similarly, multiple terms are also common with Gene Ontology as one gene can be associated with several compartments at the same time and any of these terms will be treated independently. When columns of annotation are provided, the correct number of columns must be set in the respective spin-box in the web interface (**Figure 2c**). The dataset used in the example analysis already contains three annotation columns (KEGG, Pfam and InterPro) and is available on the web site of the tool as tab separated text file and Excel file (**Figure 2a**).

Regulation or the enrichment based on various statistics (estimators) can be tested depending on the focus of the analysis (**Figure 2g**). The number of resamplings R can be set in the range from 500 to 10,000 (**Figure 2h**). The value of R states directly the resolution of the *p*-value and correlates linearly with the running time. The default value of 1,000 resamplings constitutes a compromise between accuracy and time consumption. Since the resolution of the *p*-value is limited by the number of resamplings, our tool uses interpolation between ranks of the empirical resampling distribution (*RD*) to obtain relative ranks in the interval (0, 1). In addition, optional fitting of the generalized Pareto distribution to the tails of the *RD* increases the resolution of the *p*-values and reduces the occurrence of zero *p*-values (**Figure 2j**). The minimum size (number of experimental values) of the terms *m* can be set as an absolute quantity and is defaulted to 5 (**Figure 2i**). This setting can be increased or decreased depending on the interest in rare annotations. It works as a cut off for small sample sizes and has no further impact on the analysis of samples bigger than *m*. If *m* is set to higher values, the speed of analysis increases slightly.

We recommend specifying a subject name for the analysis. The results can be received by providing an email address (**Figure 2k**). Results are displayed in three linked levels following visual information on the progress of the analysis. First, the annotation types are listed with term frequencies. Second, the corresponding terms are listed in a sortable view together with statistics and a seven-figure summary diagram [7] of the sample distribution (**Figure 3B** **)**. The seven-figure summary, similar to a box-plot, shows minima, maxima, first and third quartile and median with additional marks for the 10^th^ and 90^th^ percentile. In addition, the mean is displayed in light color. Third, the assigned proteins or genes are presented by selecting a term. A download of the complete dataset containing annotation types, terms, and statistics is available on the first level of results. To facilitate figure preparation the term view containing the diagrams can be inserted into Excel by copy-and-paste. The results are stored for a period of 14 days after generation.

## Results and Discussion

To demonstrate the usefulness of ResA we performed a pulsed stable isotope labeling experiment in living animals. Two mice were feed for two weeks with a diet containing Lys6 (purchased from Silantes). After labeling, heart tissues were isolated and extracted proteins were subjected to liquid chromatography mass spectrometry as described in [8]. RAW data were analyzed with MaxQuant (Version 2.2.9). The SILAC ratio of the labeled and unlabeled peaks (H/L) reflects the Lys6 incorporation rates of individual proteins in heart tissue. Prior to the analyses the ratios were transformed into the relative scale by H/(H+L). In addition to the GO annotation using ResA, we included KEGG, Pfam and Interpro information using the Perseus tool [9]. The dataset and the complete results are accessible on the web interface.

**Figure 4:**
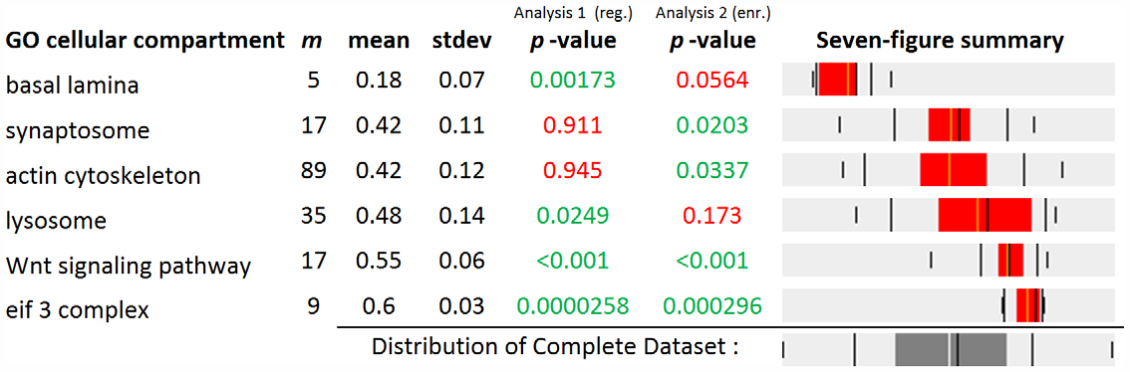
Selected Go-terms of the SILAC incorporation dataset. The mean, SD, and p-values are shown for analysis 1 (mean divided by standard deviation) and analysis 2 (interval from the 10^th^ to the 90^th^ percentile). The results are illustrates by a seven figure summary on the right site.

First, we identified annotations of proteins with significantly high or low Lys6 incorporation rates, which correspond to the detection of extremely high or low values in log_2_ fold-change distributions. For this analysis we used the mean divided by standard deviation, which is related to the t-statistic, as statistical estimator, because variations in the sample mean increase in significance when accompanied by a low standard deviation (**Figure 4** **, analysis 1**). In addition, we used the width of the interval from the 10^th^ to the 90^th^ percentile to monitor all terms with a specific narrow range of Lys6 incorporation rates regardless of their location relative to the population mean. The advantage of this non-parametric estimator over standard deviation is its robustness to outliers (**Figure 4** **, analysis 2**). The results of the analysis revealed that the protein groups with highest levels of Lys6 incorporation belong to structures such as transport vesicle, recycling endosome and eif3 complex (**Figure 4** and online results of example data). Conversely, we detected mainly GO-terms of the basal lamina in the group with low Lys6 incorporation rates showing average Lys6 incorporation of 0.18 (SD 0.07). Thus, our data confirmed recent studies in cell culture systems and living animals, which identified similar stable isotope incorporation rates for proteins in the same cellular compartments and complexes [10], [11]. Moreover, we detected the Wnt pathways with 17 members, including β-catenin, GSK3, and casein kinase with a median Lys6 incorporation of 0.55 (SD 0.06) (*p*-value <0.001) indicating very similar incorporation for these pathway members.

Clearly, the GOrilla tool is also capable to find annotations with significantly low or high Lys6 incorporation rates without explicit definition of a background set. However, this approach uses ranked lists as input and an algorithm, which is based on a minimum hypergeometric score to discover enriched GO-terms. In contrast, our approach yields complementary information to the output provided by GOrilla and the results of ResA reveal the underlying distributions of the datasets. Furthermore, ResA covers not only significant annotations but reports also non-significant terms.

In contrast to ResA the software tool ErmineJ is dedicated to the analysis of microarray data and provides a powerful resampling based gene set enrichment analysis (gene score resampling, GRS). While the annotation of ErmineJ is based on mRNA probe-set ids, ResA accepts gene symbols and Uniprot IDs for GO annotation. In cases, where a custom annotation is provided together with input data, ResA does not need a specific type of identifier. The GSR of ErmineJ works on scores representing the significance of differential gene expression such as the -log(p-value), while ResA works directly on abundance ratios or incorporation levels. Thereby, ResA retains information about the mode of regulation and provides further types of analysis using additional estimators such as SD.

**Figure 5:**
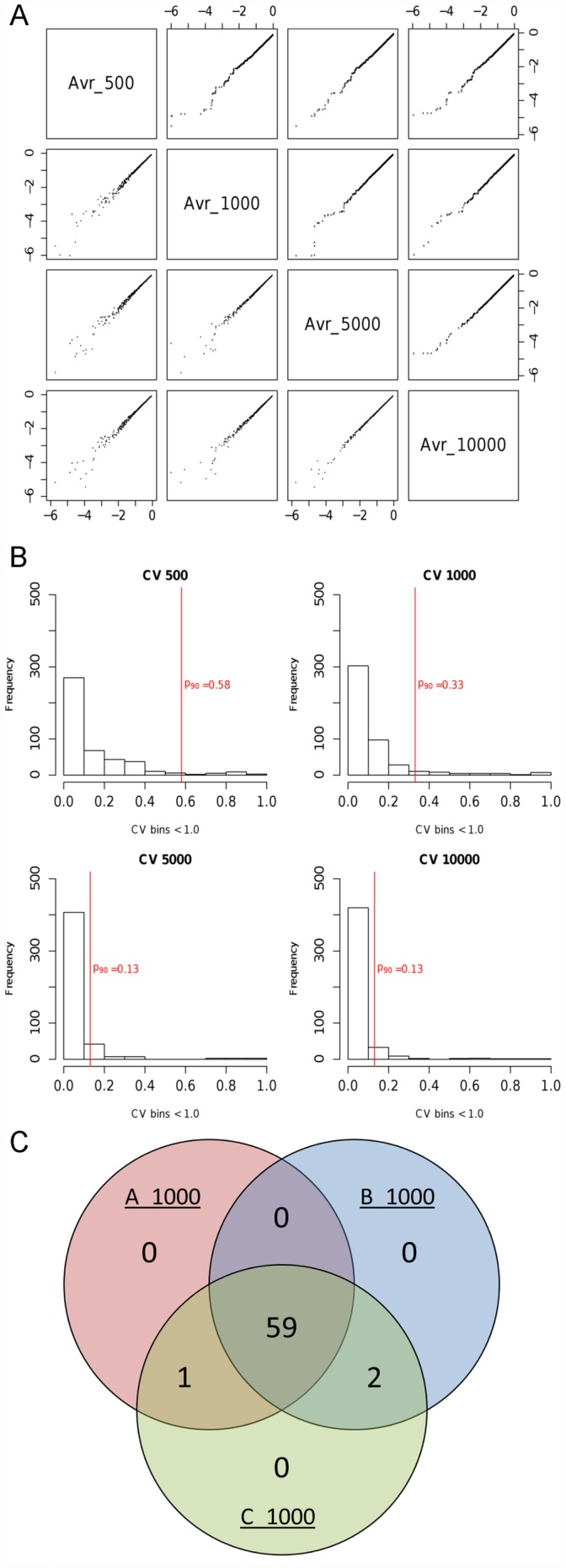
Resulting *p*-values of triplets with *R* in [500, 1000, 5000, 10000] and stability of significance. The matrix in (**A**) shows the scatterplots (lower half) and QQ-plots (upper half) of 521 triplet average *p*-values down to 10^−6^ with sampling rates (*R*) ranging from 500 to 10000. The histograms in (**B**) demonstrate the effect of different values for *R* on the coefficient of variation (CV). The vertical red lines indicate the CV at the 90^th^ percentile. The Venn diagram in (**C**) illustrates the stability of p-values for KEGG terms in a triplicate with *R* = 1000. The number of terms having a *p*-value <0.05 ranges from 60 to 62 and 59 terms (∼95%) were present in all replicates.

In order to assess the reproducibility and stability of the determined significances we used different numbers of resamplings *R* and analyzed the Lys6 incorporation datasets. The scatterplot (**Figure 5A**, lower half) and QQ-plot matrix (**Figure 5A** **,** upper half) displayed the average of the p-values for *R* equal to 500, 1000, 5000 and 10000. Plotting of the data with *R* = 500 against calculations with higher resamplings resulted in a significantly broader scatter range. However, resampling with R = 1000 resulted in *p*-values comparable to higher orders of resamples, indicating that R should be >1000 to obtain a reasonable resolution. More detailed scatterplot matrices containing all replicates and values for R between 500 and 10000 are available in **Figure S3**.

Next, we tested the correlation between calculated p-values and numbers of resamplings (R) and calculated the coefficient of variation (CV) of p-values between *R* = 500 and *R* = 10000 (**Figure 5B**). We observed higher values of the CV for *R* = 500 as compared to those with higher sampling rates, indicated by the 90^th^ percentiles (**Figure 5B**, red lines) being 0.58, 0.33, 0.13 and 0.13 for *R* equal to 500, 1000, 5000 and 10000, respectively.

The Venn diagram, **Figure 5C**, demonstrates the stability of KEGG-terms with *p*-values lower than 0.05 with *R* = 1000 in three replicates A, B and C. Of 62 different terms 59 terms were present in all replicates (>95%). Taken together, we propose *R* = 1000 as a reasonable compromise in terms of resolution, reproducibility and CPU time. In principle, due to its resampling approach, ResA is independent of sample and data set size. An exact commitment of the data set size is difficult since this property depends on the quality of the data. For typical applications, such as proteomic or transcriptomics data set analysis, sample size should not be an issue.

The combination of arbitrary annotation handling, unbiased, non-parametric empirical resampling including FDR estimation and vivid presentation of the results makes ResA a valuable tool for data analysis, which is freely available to the scientific community.

### Availability and Future Directions

The tool is available free of charge at http://resa.mpi-bn.mpg.de. The Gene Ontology annotation database (UniProt-GOA) will be updated monthly. To further increase the speed of the resampling procedure it is planned to use CUDA based parallel GPU programming.

## Supporting Information

Figure S1 **Representative Fit of the generalized Pareto distribution to the tail of the resampling distribution.** Fit of the generalized Pareto distribution to the lower 2% of the resampling distribution (*RD*) for a sample size of 11. The residual fraction of the *RD* is plotted against the corresponding residual values *x* of the RD on a logarithmic scale.
(TIF)

Figure S2 **QQ-plot of residuals of the generalized Pareto distribution fit.** QQ-plot of the residuals of the fit of the generalized Pareto distribution to the lower 2% of the resampling distribution (*RD*) for a sample size of 11.
(TIF)

Figure S3 **Detailed scatterplot matrix of triplicates for** ***R*** **in [500, 1000, 5000, 10000].** The matrix contains scatterplots of all replicates for all values of *R* against each other in the lower half and the corresponding Pearson correlation coefficients in the upper half.
(TIF)
