# Supplementary figures and images for: ResA3: A Web Tool for Resampling Analysis of Arbitrary Annotations
Source: PLoS One. 2013 Jan 28;8(1):e53743. doi: 10.1371/journal.pone.0053743 (PMC3557297; doi:10.1371/journal.pone.0053743)

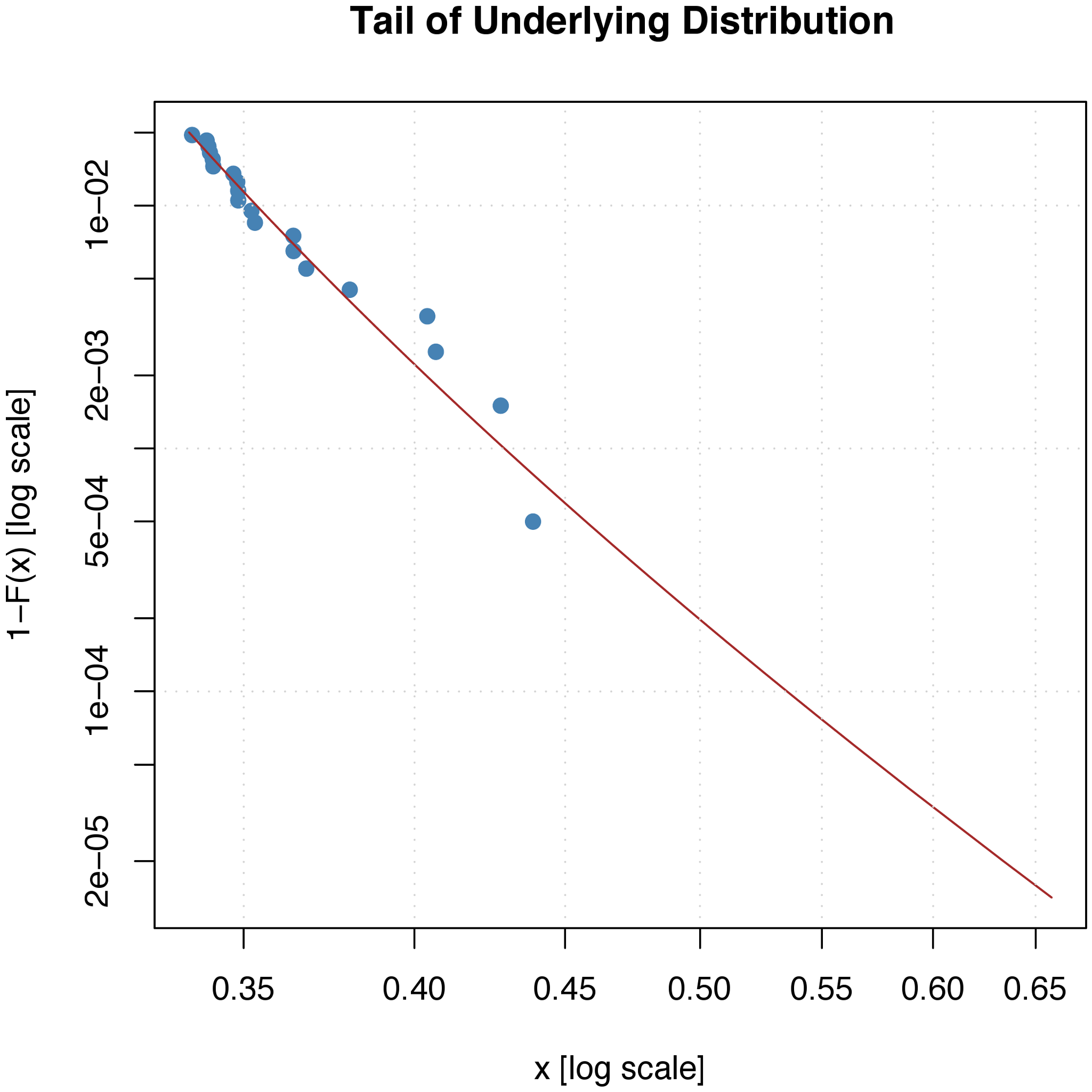

Supplement: Figure S1 — Representative Fit of the generalized Pareto distribution to the tail of the resampling distribution. Fit of the generalized Pareto distribution to the lower 2% of the resampling distribution (RD) for a sample size of 11. The residual fraction of the RD is plotted against the corresponding residual values x of the RD on a logarithmic scale. (TIF) [file pone.0053743.s001.tif]

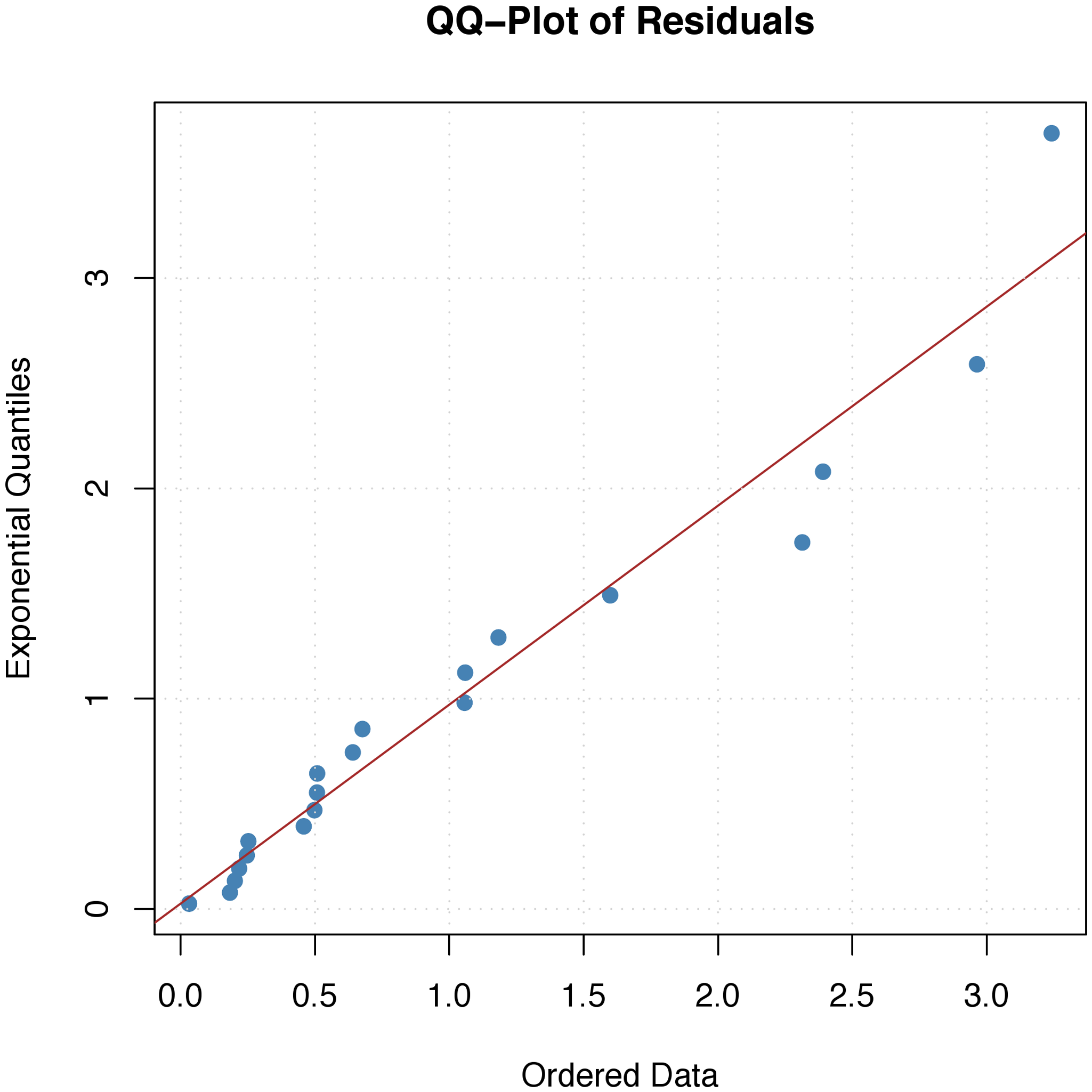

Supplement: Figure S2 — QQ-plot of residuals of the generalized Pareto distribution fit. QQ-plot of the residuals of the fit of the generalized Pareto distribution to the lower 2% of the resampling distribution (RD) for a sample size of 11. (TIF) [file pone.0053743.s002.tif]

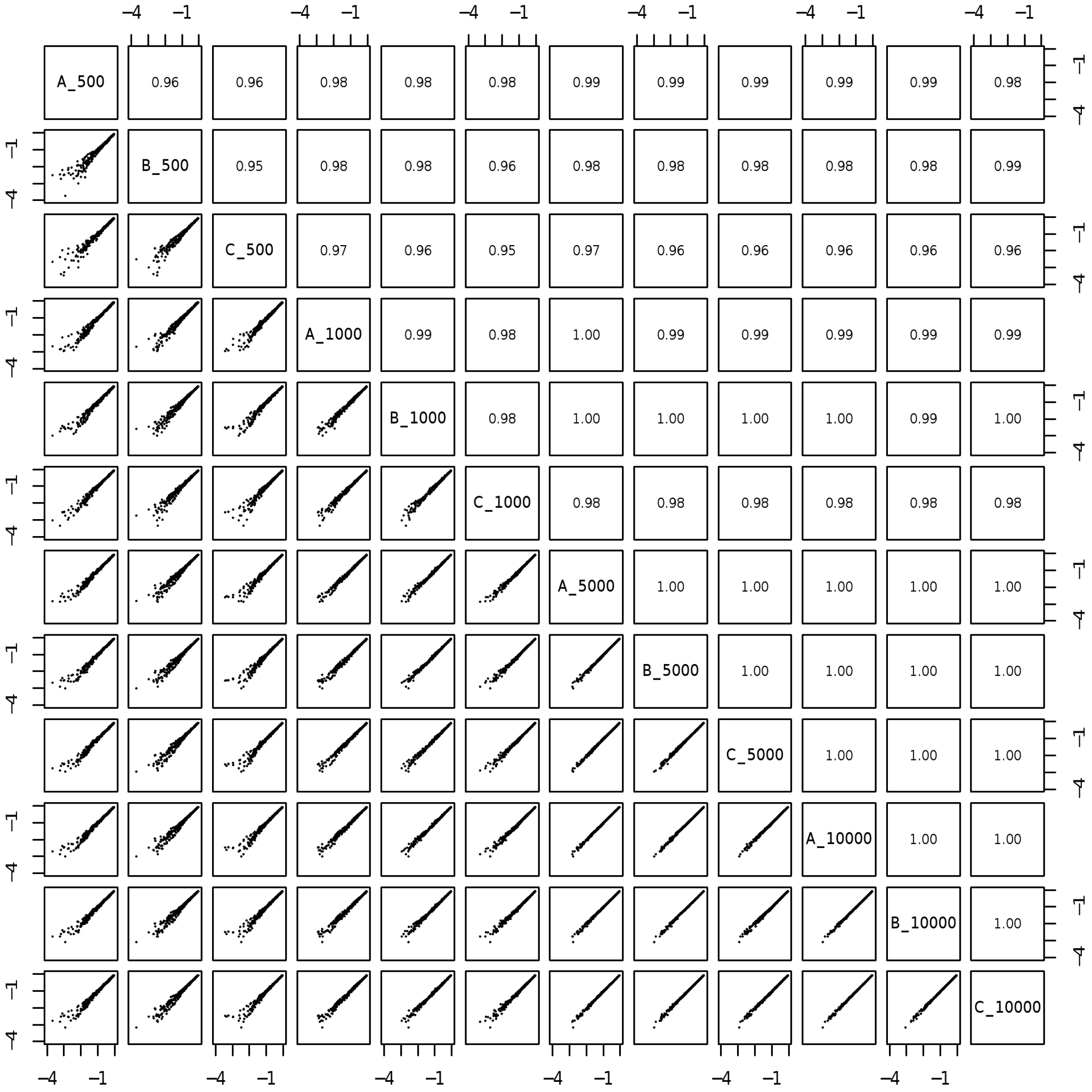

Supplement: Figure S3 — Detailed scatterplot matrix of triplicates for R in [500, 1000, 5000, 10000]. The matrix contains scatterplots of all replicates for all values of R against each other in the lower half and the corresponding Pearson correlation coefficients in the upper half. (TIF) [file pone.0053743.s003.tif]
